# Prevalence of Obstructive Sleep Apnea in the Young Adult Population: A Systematic Review

**DOI:** 10.3390/jcm13051386

**Published:** 2024-02-28

**Authors:** Katarzyna Zasadzińska-Stempniak, Hanna Zajączkiewicz, Andrzej Kukwa

**Affiliations:** Department of Otorhinolaryngology, Head and Neck Diseases, School of Medicine, University of Warmia and Mazury in Olsztyn, al. Warszawska 30, 10-082 Olsztyn, Poland; hanna.zajaczkiewicz@uwm.edu.pl (H.Z.); andrzej.kukwa@uwm.edu.pl (A.K.)

**Keywords:** sleep apnea syndrome, sleep health, obstructive sleep apnea, prevalence, polysomnography, sleep study, burden of disease

## Abstract

Background: The prevalence of obstructive sleep apnea (OSA) is suggested to differ according to different age groups. While its prevalence has been extensively investigated among middle-aged and old individuals, very few studies have summarized its prevalence among young adults. The present study aimed to conduct a systematic review and meta-analysis of OSA prevalence among healthy adults aged 18–30 years in the general population. Methods: A search of Embase, Medline, and Web of Science databases for articles reporting the prevalence of OSA among young adults confirmed by objective diagnostic methods was completed by two reviewers. Studies identified and included in the review were summarized qualitatively. Additionally, a meta-analysis of prevalence rates was conducted using a random effects model. Results: 11 articles out of 5898 met the inclusion criteria and were included in the meta-analysis. The diagnostic thresholds, scoring criteria, and the type of used device varied substantially among all the studies. We found that the pooled prevalence of OSA among young adults was 16% (CI 95%, 8–29%, I^2^ = 92%, τ^2^ = 1.47). Conclusion: The prevalence of OSA among young adults was found to be ~16%. However, a few factors diverged prevalence between the studies, such as hypopnea definition, AHI threshold, and type of device. Most of the studies included examined healthy volunteers, suggesting that the disease burden may be underestimated. Findings from our review highlight the need to include OSA-related assessment and intervention in the overall health care of young adults. By early detection and offered treatment, further complications related to comorbidities may be omitted.

## 1. Introduction

Obstructive sleep apnea (OSA) is a chronic condition defined by repeated upper airway obstruction during sleep, often associated with subsequent sudden arousals with or without oxygen desaturation [1,2]. Typical symptoms are excessive daytime sleepiness, morning headaches, poor concentration caused by recurrent nocturnal asphyxia, fatigue, nocturia, and disrupted sleep. Pathophysiological changes include significant fluctuations in blood pressure and increased sympathetic nervous system activity [1,3,4]. Untreated OSA is associated with mortality and a higher incidence of comorbidities, among them hypertension, stroke, heart failure, diabetes, and depression [5,6].

Currently, more evidence is needed to demonstrate the relative benefits of early OSA screening. In addition, studies on the natural course of OSA deterioration are scarce [7]. Therefore, early OSA diagnosis may have significant value regarding social and economic outcomes. Avoiding further complications, i.e., vehicle accidents, cardiovascular diseases, and premature death, results from early treatment of the population with a high probability of developing OSA [8,9,10].

There were several systematic reviews focusing on OSA prevalence in the general population. According to Benjafield et al., the global prevalence of OSA is almost 1 billion people affected, with the rate of OSA exceeding 50% in some countries [11,12]. Moreover, population-based studies have shown a distinct distribution of OSA in the middle-aged population, with a corresponding tendency to increase with age and growing degrees of obesity [12,13]. On the other hand, OSA is underdiagnosed due to the evolving diagnostic criteria for the condition and continuous refinement of the detection equipment [4,14]. 

Previous systematic reviews predominantly included the middle-aged population, approximately aged 30–70 years [15,16]. The data on the prevalence of OSA among young adults between 18 and 30 years are scarce. A few papers directly target the distribution of OSA among young adults [17,18,19]. However, to the best knowledge of the authors, no systematic review has focused on this subject, and we aim to address this gap by conducting a systematic review of published OSA prevalence studies among healthy adults aged 18–30 years in the general population.

## 2. Materials and Methods

This review followed guidance from Preferred Reporting Items for Systematic Reviews and Meta-Analyses (PRISMA). The study protocol is attached in Appendix A.

### 2.1. Electronic Database Search

A search of the English literature published from inception to 14.04.2022 was conducted in Embase, Medline, and Web of Science. The search terms included the following keywords and keyword combinations: sleep-disordered breathing, sleep apnea, prevalence, epidemiology, rate, and occurrence. Full search terms are included in Appendix B.

The references were exported to the EndNote citation manager (version X9.3.3) and subsequently into the Rayyan web application (https://rayyan.qcri.org) https://rayyan.ai/ (accessed on 15 February 2024) to remove the duplicates. Primary title/abstract screening was conducted by two reviewers (K.Z.S. and H.Z.) who independently evaluated and selected articles. Subsequently, studies deemed eligible were then reviewed by reading the full text and included in this systematic review according to the eligibility criteria. In case of discrepancies between reviewers, a third reviewer, an experienced specialist (A.K.) in the field, was consulted and resolved the issue. 

### 2.2. Eligibility Criteria

A study was included if the following criteria were met:

(1) Evaluating the prevalence of OSA among young adults aged 18–30 years or presenting data separately for this age subgroup;
(2) A healthy patient;
(3) OSA diagnosis was based on a polysomnographic examination or home sleep apnea testing;
(4) Published in English-language journals;
(5) Only human studies and studies conducted in English were eligible;
(6) studies based on age- or gender-specific subgroups such as studies on occupational subgroups and clinical subgroups.

The following criteria were applied to exclude not eligible studies:

(1) Reports without sufficient reporting of OSA prevalence;
(2) Narrative/systematic reviews and meta-analyses focusing on the prevalence of apnea in groups of chronic disease patients;
(3) Studies relating to the adult population without reference to age groups;
(4) Chronic disease as a requirement for inclusion in the study;
(5) Studies based solely on questionnaires.

We included cross-sectional studies and cross-sectional components of longitudinal studies in which OSA was objectively measured in adults using laboratory instruments. We also included studies based on age- or gender-specific subgroups such as studies on occupational subgroups and clinical subgroups, if age criteria were met. Studies that reported OSA in terms of the number of apnea, hypopnea, respiratory disturbance index (RDI), or respiratory event index (REI) were included. Only human studies and studies conducted in English were eligible.

Studies based solely on questionnaires were excluded. We also excluded studies on groups of patients in which the inclusion criterion was a specific disease entity.

### 2.3. Data Extraction

The following data were extracted from each eligible study: first author, year of the publication, country, design, index utilized for OSA diagnosis, the polysomnographic device specification, sample size, participants’ age, sex, body mass index (BMI), the prevalence of OSA, the apnea and hypopnea definition, OSA severity criteria. Two researchers (K.Z.S. and H.Z.) independently reviewed all eligible studies and then screened each other’s work to assure transparency. 

### 2.4. Risk of Bias Assessment of the Selected Papers

We used a risk of bias tool specifically designed to assess prevalence studies—The Risk of Bias Tool designed by Hoy et al. [20]. We rated the selected studies using this tool’s ten components assessing external validity (components 1 to 4), internal validity (components 5 to 9), and bias related to the analysis of the study (component 10). Response options for individual components were either low or high risk of bias. Each criterion was weighted equally. Each was given a score of 1 for the absence or 0 for the presence of bias. Studies with a summary score of 1 to 3 points were considered high risk of bias, between 4 and 8 points of moderate risk of bias, and 9 to 10 or more of low risk of bias.

Two reviewers (K.Z.S. and H.Z.) assessed the quality of each paper independently and their scoring was subsequently compared. A third reviewer (A.K.) led a discussion resolving any disagreement. 

### 2.5. Statistical Analysis

Extracted prevalence rates were log-transformed and combined using random effects model to derive pooled OSA prevalence rate. Results of meta-analysis were presented using a forest plot. As a secondary analysis, we stratified our analysis based on location (Europe, North and South America, Asia and Australia, Africa). The magnitude of between-study heterogeneity was approximated using I^2^ and tau statistics. All analyses were conducted in R (version 4.1.0, R Foundation for Statistical Computing, Vienna, Austria) with the use of metafor package. 

## 3. Results

### 3.1. Study Selection

**Figure 1:**
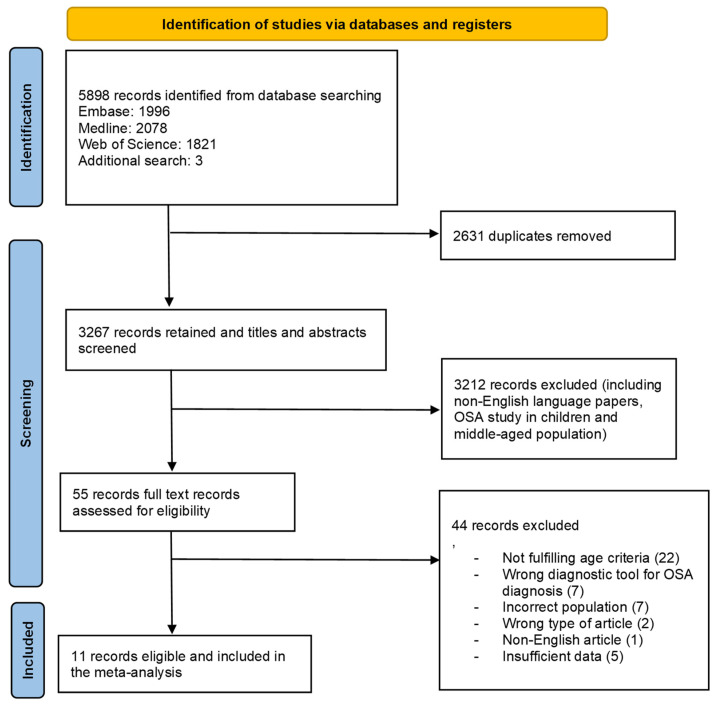
Flowchart of study screening and selection process (OSA—obstructive sleep apnea).

A total of 5898 records were retrieved from the database search. We identified 1996 articles in Embase, 2078 articles in Medline, 1821 articles in Web of Science, and three after manual search. The search strategy is presented in Appendix B. After removing the duplicates, we screened the titles and abstracts of the 3267 remaining articles. A total of 55 articles were selected for full-text review and were assessed for eligibility. Eleven articles met the inclusion criteria and were included in the meta-analysis. The flowchart of the search process and study selection is shown in Figure 1. 

### 3.2. Study Characteristics

All studies were published between 1999 and 2020. Table 1 presents the general overview of the studies included in this review. The patient’s average age was 23. The proportion of males in this sample population was 71%. Six studies comprised only a male sample [21,22,23,24,25,26]. Two studies did not report sex data [27,28]. The most frequent geographical location of the study was the USA (three studies) [22,26,28], Japan [24,29], and Australia (two studies, respectively) [18,23]. All of the studies were cross-sectional. 

Various criteria were used to diagnose OSA. The Apnea–Hypopnea Index (AHI) or Respiratory Event Index (REI) ≥ 5 was the OSA criterion in six studies [18,23,24,25,27,29]. In two of the six mentioned studies, OSA was diagnosed when presented with clinical symptoms and AHI ≥ 5 or AHI ≥ 15 without symptoms [27,29]. Another diagnostic criterion was AHI ≥ 10 [22], Respiratory Disturbance Index (RDI) ≥ 3 [30], and RDI ≥ 5 [26]. Two studies did not report the diagnostic criteria [21,28]. 

In six studies, full overnight polysomnography (PSG) was a diagnostic tool [18,21,22,25,27,28]. Home PSG with different devices (portable multichannel or single-channel device, smartwatch) was used in five studies [23,24,26,29,30].

Hypopnea assessment differed between the studies. Only three studies applied apnea and hypopnea definitions published in the American Academy of Sleep Medicine (AASM) Manual (2007 [27], 2016 [29], 2017 [18]). Three studies utilized other diagnostic criteria [25,26,30], whereas the rest did not report this information [21,22,23,24,28]. Multiple descriptions of hypopnea are presented in Appendix C.

Four studies categorized OSA severity based on an increasing AHI/REI threshold [18,22,24,25]. Two studies used indicator AHI ≥ 10 for OSA diagnosis but also extracted groups of patients with AHI < 5 and AHI 5–9 [18,22]. Two further studies distinguished groups of men and women and categorized them according to the severity gradation of OSA [18,29]. These data on OSA severity are shown in Appendix D. 

### 3.3. Risk of Bias

Overall, three studies were assessed as having a low risk of bias [18,27,28] and eight as having a moderate risk of bias [17,21,22,23,24,25,26,29]. Almost all of the studies had high ratings in external validity in three components: target population, sampling frame, and random selection. All of the studies applied validated measurement methods and had a low risk of bias in the internal validity (Appendix E).

### 3.4. Meta-Analysis of OSA Prevalence

**Figure 2:**
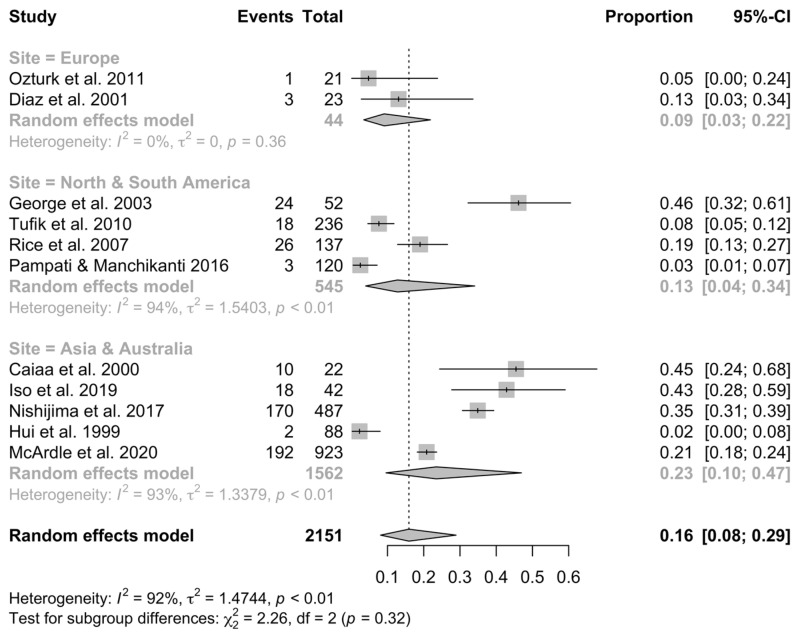
Forest plot showing the percent prevalence of OSA in young adults (CI—confidence interval).

Data were acquired from 11 studies (2151 patients from seven countries), which fulfilled the eligibility criteria. The overall pooled prevalence of OSA was 16% (CI 95%, 9–30%, I^2^ = 98%, τ^2^ = 0.94). In Asia and Australia, the prevalence was 23% (CI 95%, 10–47%, I^2^ = 93%, τ^2^ = 1.34), and in other continents, Europe was 9% (CI 95%, 3–22%, I^2^ = 0%, τ^2^ = 0) and North and South Americas were 13% (CI 95%, 4–34%, I^2^ = 94%, τ^2^ = 1.54). There was no representation of Africa due to the lack of data. Data are presented in Figure 2. 

**Figure 3:**
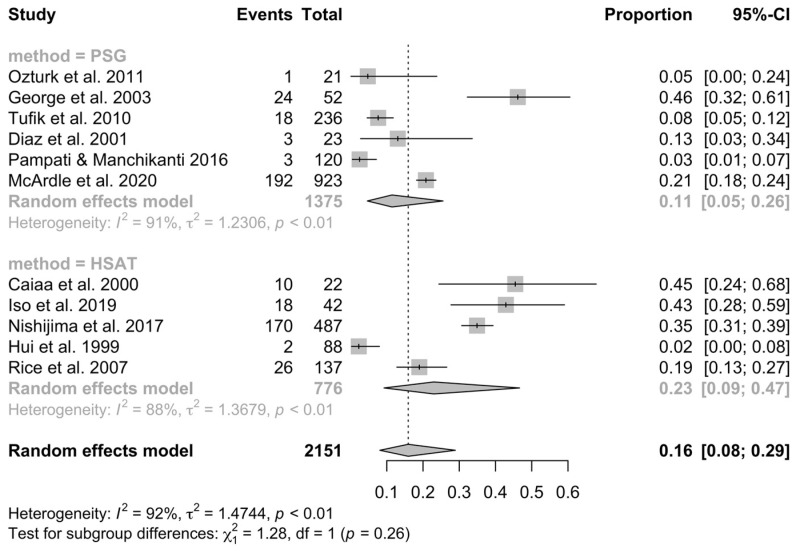
Forest plot showing the percent subgroup analysis of prevalence for different diagnostic methods of OSA in young adults (CI—confidence interval PSG—polysomnography HSAT—home sleep apnea testing).

There was no difference in prevalence between different diagnostic tools. These data are presented in Figure 3.

## 4. Discussion

The aim of this systematic review and meta-analysis was to examine the prevalence of OSA among young adults. Our work represents the most comprehensive review of evidence regarding the prevalence of OSA among young adults yet published. Accurate to April 2022, this systematic review captures the breadth of OSA reported in 11 studies, including over 2100 people. To our best knowledge, this review is among the first that exclusively included studies that relied on the objective assessment of OSA, which enhanced the reliability of the findings. In addition, we provided a pooled estimate of OSA prevalence among young adults around the globe. We found that the overall prevalence of OSA was 16%.

The overall prevalence of OSA among young adults is high, with the rate comparable to the distribution reported in the last ten years in the general adult population, ranging from 9% to 38% in a systematic review by Senaratna et al., and 3.7% to 97.3% in the systematic review by Mirrakhimov et al. [15,16]. The first mentioned review and ours included studies measuring OSA using laboratory instruments. Mirrakhimov et al. additionally included screening questionnaires as one of the possible OSA diagnostic criteria, which differs from ours and hinders comparison. Additionally, the uncertainty about the accuracy of all potential screening tools is widely known and this likely influences the pooled prevalence [31]. 

Most of the studies included in our review examined healthy volunteers, suggesting that the disease burden may be underestimated. The asymptomatic course of OSA enhances the role of screening tests for OSA. Daily symptoms are not specific, and objective recording and measurement of sleep and breathing during the night are essential for the diagnosis with objective tools [32]. Patients with OSA who have no symptoms or whose symptoms are minimally troublesome can receive preventive procedures, and exercise and weight loss can help to prevent OSA development. 

Our research provides valuable insights for healthcare professionals and policymakers, as the cost of treating the effects of OSA is a heavy burden on public health. Annually, the cost of sleep disorders is USD 130 billion, which is comparable to the cost of diabetes [33]. OSA leads to cardiovascular risks, increases car accident rates, and induces neurocognitive impairments. Moreover, it is estimated that an OSA patient costs the healthcare system about twice as much per year as a healthy patient: USD 2720 versus USD 1384 [33]. We can, therefore, expect that by diagnosing OSA sooner, its above-mentioned effects can be avoided. Knowing the rate of prevalence, the health system may prepare better for the treatment consequences of OSA. With earlier treatment implementation in younger individuals, socioeconomic trends improve, resulting in a significant decrease in sick leave and increased cognitive abilities, in summary, resulting in more efficient employees. Indeed, this is important evidence for developing an improved system for detecting OSA in young adult patients. 

The prevalence of OSA is higher in males than in females, according to data from the general population [16]. In this systematic review, generally, men were in the majority. Furthermore, there have been studies that included only a male sample [21,22,23,24,25,26]. This may indicate a problem with underreporting of OSA among the female population. Moreover, in the literature, the number of studies concerning females is significantly reduced due to reproductive age, which includes this age group. Moreover, we excluded studies concerning OSA in pregnancy due to sample variations, e.g., among others, the gestational age, weight gain, and hormone levels [34]. Additionally, females are diagnosed with OSA at older ages than males [35]. Another possible cause is a different clinical manifestation of the disease, which is perceived as a male problem [36]. Women present mild OSA with lower AHI and report various non-specific symptoms like daytime fatigue, lack of energy, insomnia, morning headaches, mood disturbances, or nightmares [35]. In this review, only four studies presented data on the severity of OSA. More severe OSA appeared in the male population. The most frequent was mild OSA with an AHI threshold of ≥5 in the male population (27–35%). 

Additionally, we observed that the criteria for the OSA diagnosis vary between the studies, thus modifying the spoken prevalence. Several factors lead to discrepancies between the reported results: hypopnea definition, AHI threshold, and type of device. Firstly, the hypopnea definition was modified throughout the years and may result in a different number of the total AHI [37,38,39,40,41]. In our review, only three studies used the AASM hypopnea definition (2007, 2016, 2017). Three studies utilized other diagnostic criteria, whereas the rest did not report this information. The 2007 AASM recommendations published two hypopnea definitions: recommended and alternative, depending on the sensors used [42]. The recommended hypopnea definition is based on a 30% or more reduction in nasal pressure signal associated with ≥4% desaturation. The alternative hypopnea definition needs a 50% or more reduction in nasal pressure signal associated with ≥3% desaturation or arousal. The 2012 AASM hypopnea definition requires a decrease in airflow of ≥30% (by a valid measure of airflow) lasting ≥10 s, associated with either ≥3% desaturation from the pre-event baseline or arousal. Bahammam et al. compared three hypopnea definitions from different guidelines. The recommended definition resulted in lower AHI than the AASM 2007 alternative and AASM 2012 [38]. The 2012 AASM definition increased the AHI number, which was also confirmed by Duce et al. [39]. Thus, that may lead to the underdiagnosing of OSA, and a lower prevalence of OSA, when the AASM 2007 hypopnea definition is used.

Secondly, the scoring criteria are a further reason for only a partially feasible comparison of the prevalence. AHI is the number of apnea and hypopnea for the hour of sleep. Generally, the AHI threshold to diagnose OSA was ≥5, which is in accordance with AASM’s recent recommendations [43]. However, in one study with full overnight PSG, the AHI score was ≥10 [22]. Moreover, another index was used at home PSG: RDI or REI. RDI is the number of apnea, hypopnea, and respiratory effort-related arousals for total sleep time. REI is the number of apnea and hypopnea for the monitoring time [44]. The diagnostic thresholds varied among all these studies. The heterogeneity of applied units and levels indicates the importance of structuring and standardizing the indicators used in home PSG and full overnight PSG. This unification is a desirable ongoing process and is reflected in subsequent guidelines.

Lastly, the type of used device influences the comparison of the results. There was no difference in prevalence between different diagnostic tools. Traditionally, we distinguish four types of diagnostic devices. Type 1: the full overnight PSG is a gold standard for OSA diagnosis and records at least seven channels. Type 1 was applied in more than half of the studies included in this systemic review, which makes the studies comparable and reduces the likelihood of diagnostic error. The rest of the studies used other types, 2, 3, and 4, which are for home sleep apnea testing (HSAT). The difference between them is in the number and type of sensors used, with type 4 devices recording only one sensor. The detected parameters vary between the HSAT devices and are not unified, so the diagnosis of OSA likely may not be comparable. However, the diagnostic potential for OSA is high as it is more approachable than in-laboratory PSG [45]. The benefit of HSAT is that the test may be performed at home, which is the closest condition to a natural sleep pattern. They do, though, have their disadvantages. Firstly, the total sleep time cannot be recorded—it reports the time of total recording. In addition, they do not detect awakenings and unaware arousals. This contributes to the high number of false-negative results. Thus, we can suspect that in the meta-analyzed studies that utilized HSAT, the prevalence of OSA in young adults may be higher. Nowadays, recommendations for OSA diagnosis enable HSAT and full overnight PSG for OSA diagnosis, with the enlightenment of HSATs’ potential disadvantages. The AASM recommends the use of HSAT for the diagnosis of OSA in selected populations but advises against its use as a population screening tool [43].

Additionally, an important finding of this review is the regional differences in OSA prevalence. The prevalence of OSA in Asia and Australia is higher than in North and South America and Europe. However, there is no data from Africa, a major part of Asia and Europe. There is a need for similar studies in those blind regions.

Our review is not devoid of limitations. First, although we did an exhaustive search, not all the literature was captured in this review, such as papers published in non-English. Second, the women population was under-represented. That is the result of a higher prevalence of OSA in males until they reach 70 years old. Therefore, findings from this study may not be generalized to young women. It is also worth mentioning that the majority of the studies included in this systematic review had a moderate risk of bias for the estimate of OSA prevalence. Moreover, there is a deficiency of reproducible cross-sectional studies, which would allow monitoring trends in the prevalence of OSA in different localizations. Furthermore, the representativeness of the sample population is another limitation. The analyzed studies are small, most of them hospital-based, which increases the risk of oversampling, i.e., detecting more OSA patients than in the general population.

An undoubtful strength of this study lies in the diagnosis of OSA using objective methods. We determined the prevalence of OSA in different regions. We have shown that different hypopnea definitions, AHI thresholds, and devices influence the prevalence. 

## 5. Conclusions

This meta-analysis revealed that the prevalence of OSA in young adults is 16%. We pointed main factors diverging prevalence between the studies, such as hypopnea definition, AHI threshold, and type of device. Findings from our review highlight the need to include OSA-related assessment and intervention in the overall health care of young adults. By early detection and offered treatment, further complications of OSA related to comorbidities may be omitted.

## Figures and Tables

**Table 1:** Summary of studies included in the review.

Author	Year	Country	Design	Index Utilized for OSA Diagnosis	Diagnostic Tool	Population Size	Age (Years)	BMI	Sex (% Male)	Number of Patients with OSA
Ozturk et al.	2011	Turkey	Cross-sectional study	NR	Full overnight PSG: one night at the sleep laboratory for adaptation before the experimental measurements (3 nights)	21	18–24	NR	100%	0
George et al.	2003	USA	Cross-sectional study	AHI ≥ 10	Full overnight PSG	52	25.5 ± 2.7	31.5 ± 4.6	100%	24
Caiaa et al.	2020	Australia	Cross-sectional study	AHI ≥ 5	Home PSG: portable multi-channel device (Alice PDX, Philips Respironics, OR, USA)	22	23.8 ± 3.6	30.0 ± 2.2	100%	10
Tufik et al.	2010	Brazil	Cross-sectional study	AHI ≥ 5 and clinical symptoms and or AHI ≥ 15	Full overnight PSG: a digital system at the sleep laboratory (EMBLA S7000, Embla Systems, Inc., Broomfield, CO, USA)	236	20–29	NR	NR	18
Iso et al.	2019	Japan	Cross-sectional study	AHI ≥ 5 events/1 h over a total sleeping time of more than 3 h	Home PSG: WatchPAT-200 device- a validated surrogate for PSG (Itamar Medical Ltd., Caesarea, Israel)	42	18.6 ± 0.5	27.7 ± 4.0	100%	18
Diaz et al.	2001	Spain	Cross-sectional study	AHI ≥ 5	Full overnight PSG (Somnostar 4100, Sensor Medics, San Rafael, CA, USA, EE.UU.)	23	20–29	NR	100%	3
Nishijima et al.	2017	Japan	Cross-sectional study	REI ≥ 5 and clinical symptoms and or REI ≥ 15 without clinical symptoms. REI is calculated from the number of total respiratory events divided by monitoring time	Home PSG: type 3 portable monitors (smartwatch PMP 300E Philips Respironics GK)	487	24.6 ± 1.9	22.3 ± 3.2	74%	170
Hui et al.	1999	China	Cross-sectional study	RDI ≥ 3	Home PSG: MESAM IV device (4-channel device)	88	20.7 ± 2.3	20 ± 2.3	50%	2
Rice et al.	2007	USA	Cross-sectional study	RDI ≥ 5	Home PSG: A single-channel screening tool measures airflow using a nasal cannula with a pressure transducer (ApneaLinkTM (AL; ResMed Corp.Poway, CA)	137	27 ± 3	32.4 ± 4	100%	26
Pampati et al.	2016	USA	Cross-sectional study	NR	Accredited sleep laboratory	120	18–24.9	NR	NR	3
McArdle et al.	2020	Australia	Cross-sectional study	AHI ≥ 5	Full overnight PSG- level 1 n-laboratory PSG study in a bedroom-like environment (GRAEL, Compumedics)	923	22.2	NR	49%	192

NR—not reported; PSG—polysomnography; AHI—Apnea–Hypopnea Index; REI—Respiratory Event Index; RDI—Respiratory Disturbance Index; AASM—American Academy of Sleep Medicine.

**Figure A1:**
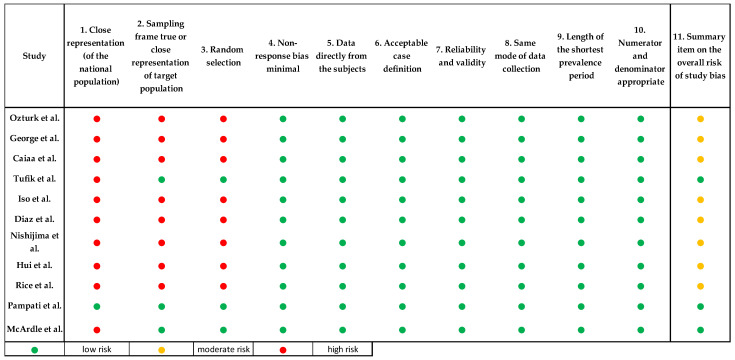
Risk of bias assessment.

## Data Availability

The data presented in this study are available on request from the corresponding author. The data are not publicly available due to the protection of personal data.

## Appendix A

**Study protocol for prevalence of obstructive sleep apnea in the young adult population**

Study Information Introduction: Obstructive sleep apnea (OSA) is a chronic condition defined by repeated upper airway obstruction during sleep, often associated with subsequent sudden arousals with or without oxygen desaturation [1,2]. Typical symptoms are excessive daytime sleepiness, morning headaches, poor concentration caused by recurrent nocturnal asphyxia, fatigue, nocturia, and disrupted sleep. Pathophysiological changes include significant fluctuations in blood pressure and increased sympathetic nervous system activity [1,3,4]. Untreated OSA is associated with mortality and a higher incidence of comorbidities, among them hypertension, stroke, heart failure, diabetes, and depression [5,6].

Justification: Currently, more evidence is needed to demonstrate the relative benefits of early OSA screening. In addition, studies on the natural course of OSA deterioration are scarce [7]. Therefore, early OSA diagnosis may have significant value regarding social and economic outcomes. Avoiding further complications, i.e., vehicle accidents, cardiovascular diseases, and premature death, results in early treatment of the population with a high probability of developing OSA [8,9,10]. According to Benjafield et al., the global prevalence of OSA is almost 1 billion people affected, with the rate of OSA exceeding 50% in some countries [11,12]. Moreover, population-based studies have shown a distinct distribution of OSA in the middle-aged population, with a corresponding tendency to increase with age and growing degrees of obesity [12,13]. On the other hand, OSA is underdiagnosed due to the evolving diagnostic criteria for the condition and continuous refinement of the detection equipment [4,14]. Considering that there were several systematic reviews focusing on OSA prevalence in the general population, we did not identify any regarding the group of young adults. Previous systematic reviews predominantly included the middle-aged population, approximately aged 30–70 years [15,16]. The data on the prevalence of OSA among young adults between 18 and 30 years are scarce. A few papers directly target the distribution of OSA among young adults [17,18,19]. However, to the best knowledge of the authors, no systematic review has focused on this subject, and we aim to address this gap by conducting a systematic review of published OSA prevalence studies among healthy adults aged 18–30 years in the general population.

Design Plan

Study type Meta-Analysis—A systematic review of published studies.

Blinding No blinding is involved in this study.

Is there any additional blinding in this study? Not applicable

Study design

For the first objective, we will collect data on the prevalence of OSA in the young adult population.

For the second objective, we will conduct a systematic review.

We will conduct a systematic search of the available evidence on the prevalence of obstructive sleep apnea in the young adult population.

**Inclusion criteria** *Type of studies:* We will include observational studies (longitudinal and cross-sectional) evaluating the prevalence of OSA among young adults aged 18–30 years or presenting data separately for this age subgroup including those embedded in randomized controlled trials. We will exclude case reports and case series.

*Type of participants:* We will include healthy young adults aged 18–30 years.

*Type of outcome measures:* OSA diagnosis was based on a polysomnographic examination or home sleep apnea testing.

Sample size: Not applicable.

Sample size rationale: *No response.*

Stopping rule: *No response.*

**Search methods for identification of studies:** We will perform a comprehensive, systematic search with no restrictions on the language of publication or publication status. We will search the following sources from the inception of each database: Embase, Medline, and Web of Science. We constructed an empirically derived search strategy.

**Searching other resources:** We will try to identify other potentially eligible trials or ancillary publications by searching the reference lists of retrieved included trials.

**Analysis Plan**

**Objective 1: Data charting**

**Data collection and analysis, selection of studies**: We will use EndNote for deduplication and Rayyan for study selection. Two review authors (K.Z.S. and H.Z.) will independently scan the abstract, title, or both of the remaining records retrieved to determine which studies should be assessed further through Rayyan. Two review authors (K.Z.S. and H.Z.) will investigate all potentially relevant records as full text and classify studies as included studies, excluded studies, and studies awaiting classification, following the criteria. We will resolve any discrepancies through consensus or recourse to a third review author (AK).

**We will extract data**: First author, year of the publication, country, design, index utilized for OSA diagnosis, the polysomnographic device specification, sample size, participants’ age, sex, body mass index (BMI), the prevalence of OSA, the apnea and hypopnea definition, OSA severity criteria. Two researchers (K.Z.S. and H.Z.) will independently review all eligible studies and then screen each other’s work to ensure transparency.

**Objective 2: Data synthesis**

We anticipate clinical and methodological heterogeneity in the body of research. We will aim to produce an interim report in the form of a systematic review. When there is sufficient homogeneity across the study population, diagnostic approaches, and measurements of outcomes, we will summarize data using meta-analysis. We will not pool data from different study designs (cohorts and cross-sectional studies). We will interpret meta-analyses with due consideration. We will use a model for pooling proportions using R (version 4.1.0, R Foundation for Statistical Computing, Vienna, Austria) with the use of a metaphor package that allows the computation of 95% confidence intervals.

**Assessment of heterogeneity**

We will evaluate the percentage of total variation across studies due to heterogeneity by the I^2^ measure. We will use the thresholds “low” (0–40%), “moderate” (30–60%), “substantial” (50–90%), and considerable (75–100%) following the Cochrane Handbook [25]. As we expect that heterogeneity will be high, we aim to explore heterogeneity by analyzing our pre-specified subgroups (see below)

**Publication bias**

We will evaluate publication bias by using The Risk of Bias Tool designed by Hoy et al.

Conflicts of Interest

None

Funding sources

Not applicable

## Appendix B

**Table A1:** Search strategy summary.

Data Base	Strategy	Results 15 February 2024
EMBASE	(((‘sleep apnea’ OR ‘sleep disordered breathing’) NEAR/3 (‘epidemiology’ OR ‘rate’ OR ‘prevalence’ OR ‘occurrence’)):ti,ab) AND [humans]/lim AND [embase]/lim	1996
MEDLINE	TI=((“sleep disordered breathing” OR “sleep apnea”) NEAR/3 (“prevalence” OR “epidemiology” OR “rate” OR “occurrence”)) OR AB=((“sleep disordered breathing” OR “sleep apnea”) NEAR/3 (“prevalence” OR “epidemiology” OR “rate” OR “occurrence”))	2078
WOS	TI=((“sleep disordered breathing” OR “sleep apnea”) NEAR/3 (“prevalence” OR “epidemiology” OR “rate” OR “occurrence”)) OR AB=((“sleep disordered breathing” OR “sleep apnea”) NEAR/3 (“prevalence” OR “epidemiology” OR “rate” OR “occurrence”))	1821

## Appendix C

**Table A2:** Multiple definitions of hypopnea in the studies included in the review.

Author	Apnea and Hypopnea Definition
Ozturk et al.	NR
George et al.	NR
Caiaa et al.	NR
Tufik et al.	AASM 2007
Iso et al.	NR
Diaz et al.	Apnea was defined as the cessation of airflow for ≥10 s. Hypopnea was defined as a decrease in airflow or toracoabdominal movements by ≥50% for 10 s, accompanied by oxygen desaturation ≥ 4% or microarousal
Nishijima et al.	AASM 2016
Hui et al.	Apnea was defined as the cessation of airflow for ≥10 s, and hypopnea was defined as a reduction of airflow ≥50% for ≥10 s.
Rice et al.	Apnea was defined as a decrease in airflow of 80% of more from baseline for at least 10 s. Hypopnea was defined as a decrease in airflow by 50% to 80% of baseline for ≥10 s.
Pampati et al.	NR
McArdle et al.	AASM 2017

Abbreviations: AASM—American Academy of Sleep Medicine.

## Appendix D

**Table A3:** OSA severity in the studies included in the review.

		Sample Size		AHI 5-9,9	AHI 10<	
George et al. 2003	All (Male)	52		10	14	
Diaz et al. 2001	All (Male)	23		1	2	
		Sample size	REI 5≤	low REI 5–15	moderate REI 15–30	high REI 30≤
Nishijima et al. 2017	All	487	170	141	19	10
	Male	358	158	129	19	10
	Female	129	12	12	0	0
			AHI 5≤	AHI 5≤ AND ESS 11≤	AHI 15≤	
McArdle et al. 2020	All	923	192	26	34	
	Male	454	122	17	22	
	Female	469	70	9	12	

Abbreviations: AHI—Apnea–Hypopnea Index; REI—Respiratory Event Index.
